# Correlation between seminal plasma biochemical markers and semen parameters in idiopathic oligoasthenoteratospermia: identification of biomarkers for L-carnitine therapy

**DOI:** 10.3389/fendo.2024.1330629

**Published:** 2024-03-12

**Authors:** Qilong Yuan, Ruifang Hong, Yunping Ni, Manbo Jiang, Juan Liu, Zhiqiang Chen, Dongyu Yang

**Affiliations:** ^1^ Department of Reproductive Medicine, Guangdong Provincial Hospital of Chinese Medicine, Guangzhou, China; ^2^ Department of Pharmacy, Guangdong Provincial Fertility Hospital, Guangzhou, China

**Keywords:** seminal plasma biochemical markers, semen parameters, L-carnitine, male infertility, oligoasthenoteratospermia

## Abstract

**Background:**

L-carnitine therapy for idiopathic sperm abnormalities exhibits variable effectiveness, and currently, there are no established criteria to predict patient response. This study investigated correlations between seminal plasma markers and semen parameters to identify biomarkers that can guide indications for L-carnitine therapy indications in patients with idiopathic sperm abnormalities.

**Methods:**

A retrospective review was conducted on 223 male patients with idiopathic oligoasthenoteratospermia, who sought medical attention at our clinic between January 2020 and October 2022. These patients underwent a pretreatment seminal plasma biochemical analysis, followed by a three-month continuous L-carnitine treatment. The correlation between seminal plasma biochemical parameters and pretreatment semen parameters was analyzed. Semen quality was compared between cases with normal and abnormal seminal plasma biochemical parameters, both pretreatment and posttreatment. The correlation between the changes in semen parameters after treatment and seminal plasma biochemical parameters were investigated.

**Results:**

Correlation analyses revealed significant associations between all pretreatment semen parameters and seminal plasma biochemical markers, except for liquefying time and the ratio of normal morphology. Subgroup analysis, stratified by seminal fructose, zinc, citric acid, and neutral glycosidase levels, demonstrated that abnormal groups exhibited significantly different levels of semen parameters compared with the normal groups. The changing difference and changing ratio in the ratio of forward motile sperm showed a negative correlation with seminal fructose levels (r=-0.165 and -0.144). The changing difference in semen volume was negatively correlated with the level of seminal neutral glycosidase (r=-0.158). The changing ratio in semen volume, sperm concentration, total sperm count, and count of forward motile sperm all exhibited negative correlations with the levels of seminal neutral glycosidase (range from -0.178 to -0.224).

**Conclusion:**

Seminal plasma biochemical markers, particularly fructose and neutral glycosidase, may serve as valuable indicators for determining the eligibility of patients with idiopathic sperm abnormalities for L-carnitine therapy.

## Introduction

Idiopathic sperm abnormalities represent a prevalent condition impacting male fertility, characterized by low sperm count, low sperm motility, or abnormal morphology without discernible pathological causes (1). Spermatogenesis, a complex process influenced by numerous internal and external factors (2), often results in an idiopathic diagnosis of infertility, prompting the widespread adoption of empirical and comprehensive treatments. The recognition of oxidative stress as a contributing factor to sperm damage (3) has spurred the utilization of antioxidant medications, such as L-carnitine, either as standalone therapies or in combination with other drugs, for the prolonged management of idiopathic sperm abnormalities (4, 5).

Nevertheless, despite a reasonably well-defined mechanism of drug action, the therapeutic effectiveness of antioxidants in treating idiopathic sperm abnormalities exhibits considerable variability (6–8). Furthermore, there is a lack of evidence-based criteria for discerning patients likely to derive benefit from this treatment (9–11). Addressing these challenges requires prompt efforts to pinpoint the appropriate patient population with idiopathic sperm abnormalities for antioxidant drug treatments.

In an animal study involving Teddy (Capra hircus) goats, Umar et al. illustrated dynamic interactions among different components of semen quality measurements and seminal plasma biochemical markers (12). In clinical settings, seminal plasma biochemical markers are frequently employed to evaluate the functional status of the epididymis and other accessory glands (13). However, the variability in detection methods and the broad spectrum of reference values may result in the inaccurate interpretation of the clinically significant reference values for these markers (14, 15).

To identify biomarkers that can be utilized to establish indications for L-carnitine therapy in patients with idiopathic sperm abnormalities, this study aimed to investigate the correlation between seminal plasma biochemical markers and semen parameters. The identification of pertinent biomarkers has the potential to enhance the accuracy of treatment selection and improve the effectiveness of L-carnitine therapy, thereby playing a crucial role in the effective management of infertility in this population.

## Materials and methods

### Study design and participants

This research was a single-center, retrospective cohort study conducted at a reproductive medicine center. From January 2020 to October 2022, 223 male outpatients seeking assistance for infertility concerns at the center were included. The inclusion criteria were as follows: (1) patients aged between 23 and 45 years; (2) a history of infertility lasting for more than one year; (3) normal testicular volume (with each testis measuring between 12-18 ml) and normal sex hormone levels; and (4) semen parameters following the 5th edition of the World Health Organization (WHO) laboratory manual. Patients were excluded from the study if they met any of the following criteria: (1) elevated semen white blood cell counts and abnormal liquefaction, (2) recent use of medications known to affect semen quality during the intervention period, (3) presence of underlying factors affecting sperm quality, such as varicocele, infections, and trauma. This study was approved by the Guangdong Provincial Hospital of Chinese Medicine Research Ethics Committee (Approval No: ZE2023-157-01) and was performed in accordance with the Declaration of Helsinki. The ethics committee waived the requirement of written informed consent for participation from the participants or the participants’ legal guardians/next of kin because of the retrospective nature of the study.

### L-carnitine treatment

All patients received oral administration of levocarnitine solution (Northeast Pharmaceutical Group Shenyang First Pharmaceutical Co., Ltd., 10 ml:1 g/vial, lot number 191202-210943) at a dose of 1 vial per administration, twice daily, for a continuous period of three months. All patients received guidance on lifestyle adjustments aimed at optimizing treatment efficacy. These adjustments encompassed maintaining regular sleep patterns, avoiding late nights, abstaining from alcohol and tobacco, and avoiding chronic exposure to heat (including fever), radiation, and toxins. All patients were explicitly informed of these precautions, and instances of special circumstances were documented and assessed by the medical professionals to determine their inclusion in the analysis.

### Assessment of semen parameters and seminal plasma biochemical markers

Semen routine and seminal plasma biochemical analysis were performed in accordance with the WHO’s laboratory manual for the examination and processing of human semen (5th edition) (16). Semen samples were collected through masturbation into sterile and dry containers following a sexual abstinence period of 2-7 days. Sperm quality was assessed utilizing the Spanish SCA automatic semen analysis system, Nikon 50i phase-contrast microscope, and Siemens Bayer ADVIA Centaur automated chemistry and immunoassay analyzer. The analyzed semen parameters included semen volume, sperm concentration, total sperm count, motility, morphology, semen white cell levels, and liquefying time. The seminal plasma biochemical markers included fructose, zinc, citric acid, and neutral glycosidase.

Sperm morphology was assessed using an improved Papanicolaou staining method. Slides were scrutinized under an oil immersion objective at 1000x magnification, with a minimum of 200 spermatozoa counted consecutively. Semen white blood cell concentration was determined using the Semen Leukocytes Peroxidase Staining Kit (Peroxidase Staining, SemenAssay^®^, BRED Life Science Technology Inc., Shenzhen, China). Levels of seminal plasma neutral alpha-glucosidase (SemenAssay^®^ NAG, Modified Cooper method) and citric acid (SemenAssay^®^ Citric, Enzymatic method) were assessed using test kits from BRED Life Science Technology Inc. Based on biochemical analyses of seminal plasma from fertile men, the normal reference ranges were established using the 5th percentile. The WHO recommends a normal reference value for neutral α-glucosidase detection of ≥20 mU/ejaculation (16). For citric acid detection, the recommended normal reference value is 52 μmol/per time (17). Levels of seminal plasma zinc and fructose were determined by using chemical colorimetric test kits obtained from Nanjing Xindi Bio-Pharmaceutical Engineering Co., Ltd. (Nanjing, China). According to the Human Sperm Bank Technical Training of the National Health Commission of China, the recommended reference values for seminal fructose using the resorcinol method are 0.87 to 3.95 g/L, and for seminal zinc using the chemical colorimetric method, the normal reference range is 0.8 to 2.5 mmol/L (18). The choice of these methods is based on the increasing trend in reported correlations between seminal fructose and zinc concentrations with seminal parameters (19, 20). The aforementioned seminal plasma biochemical markers were analyzed using an automated biochemical analyzer.

### Statistical analysis

Continuous variables are presented as the mean ± standard deviation (SD) and median. The comparison of means between groups was conducted using Student’s independent t test or Mann–Whitney U test (if normality assumptions were not met). The comparisons between pretreatment and posttreatment results were performed using Student’s paired t-test or the Wilcoxon signed-rank test. Pearson’s correlation coefficient was employed to depict the relationships between continuous variables. Multiple linear regression was utilized to further confirm the change in semen parameters after treatment with adjustments made for patient fructose, zinc in seminal plasma, citric acid, neutral glycosidase levels, and age. All analyses were performed using IBM SPSS Version 25 (SPSS Statistics V25, IBM Corporation, Somers, New York). The statistical significance level for all the tests was set at a two-tailed P value < 0.05.

## Results

### Patient clinical characteristics

A total of 223 male patients, with an average age of 33.41 ± 4.53 (median 33) years were included in this study. **Table 1** presents the patient’s demographic and clinical characteristics, including age and seminal plasma biochemical markers (fructose, zinc, citric acid, neutral glycosidase), along with pretreatment and posttreatment semen parameters. These parameters encompass abstinence days, the ratio of forward motile sperm (%), semen volume (ml), sperm concentration (million/ml), white cell count (million/ml), liquefying time (minute), ratio of normal morphology (%), total sperm count (million), and count of forward motile sperm (million). As indicated in **Table 1**, following treatment, a significant improvement was observed in the semen parameters, including the ratio of forward motile sperm (%), semen volume (ml), sperm concentration (million/ml), ratio of normal morphology (%), total sperm count (million), and count of forward motile sperm (million) (all P<0.05).

**Table 1:** Clinical characteristics of all patients, mean ± SD (median).

Parameters	Pretreatment (baseline)	Posttreatment	P
Basic information			
Age, year	33.41 ± 4.53 (33.00)		
Seminal plasma biochemical markers			
Fructose, g/L	2.01 ± 1.08 (2.00)		
Zinc in seminal plasma, mmol/L	2.45 ± 0.98 (2.42)		
Citric acid, umol/per time	103.80 ± 58.10 (92.26)		
Neutral glycosidase, mU/per time	61.03 ± 41.48 (55.44)		
Semen parameters			
Abstinence, day	4.13 ± 1.93 (4.00)	4.15 ± 1.27 (4.00)	0.444
Ratio of forward motile sperm, %	24.67 ± 12.56 (23.70)	30.50 ± 13.54 (29.60)	<0.001
Semen volume, ml	3.32 ± 1.34 (3.20)	3.54 ± 1.41 (3.40)	0.001
Sperm concentration, million/ml	59.58 ± 62.77 (45.70)	68.33 ± 78.77 (47.50)	0.012
White cell, million/ml	0.46 ± 0.93 (0.20)	0.42 ± 0.75 (0.20)	0.625
Liquefying time, minute	29.71 ± 5.53 (30.00)	30.49 ± 5.83 (30)	0.082
Ratio of normal morphology, %	2.65 ± 1.64 (2.00)	3.11 ± 1.81 (3.00)	<0.001
Total sperm count, million	173.95 ± 130.46 (139.05)	207.14 ± 170.89(162.36)	0.007
Count of forward motile sperm, million	42.13 ± 38.41 (31.89)	61.11 ± 53.76 (49.46)	<0.001

The comparisons between pre- and post-treatment were using Wilcoxon signed ranks test.

### Correlations between pretreatment semen parameters and seminal plasma biochemical markers

To examine the relationship between pretreatment semen parameters and seminal plasma biochemical markers, correlation coefficient analyses were performed. As shown in **Table 2**, all pretreatment semen parameters exhibited significant correlations with some or all of the seminal plasma biochemical markers, except for the ratio of normal morphology. These results suggest that seminal plasma biochemical markers may exert an influence on baseline seminal parameters. To gain further insights into these influences, subsequent subgroup analyses were undertaken.

**Table 2:** Correlation coefficient analysis between pretreatment semen parameters and seminal plasma biochemical markers.

Pearson’s r	Fructose	Zinc in seminal plasma	Citric acid	Neutral glycosidase
Age, year	-0.029	0.106	-0.001	0.020
Pretreatment semen parameters				
Abstinence, day	-0.062	0.067	0.125*	0.062
Ratio of forward motile sperm, %	0.275**	-0.201**	0.022	-0.053
Semen volume, ml	0.392**	-0.231**	0.543**	0.325**
Sperm concentration, million/ml	-0.344**	0.152**	-0.137*	0.155**
Ratio of normal morphology, %	-0.097	0.014	-0.029	-0.052
Total sperm count, million	-0.178**	0.044	0.106	0.395**
Count of forward motile sperm, million	0.031	-0.067	0.140*	0.299**

The association between variables was analyzed using Pearson’s correlation coefficient.

*P<0.05; **P<0.01.

### Subgroup analyses stratified by seminal fructose and zinc levels

All variables were compared between the abnormal (<0. 87 g/L) and normal (≥0.87 g/L) fructose groups. As shown in **Table 3**, the abnormal group exhibited significantly higher levels of zinc in seminal plasma, a longer abstinence day (pre), higher sperm concentration (pre and post), and lower ratio of forward motile sperm (pre) and semen volume (pre and post) (all *P*<0.05) compared to the normal group. Notably, all abstinence periods ranged from 2 to 7 days, both before and after treatment, in compliance with the specified regulation. Before treatment, the normal group demonstrated a significantly higher ratio of forward motile sperm (*P*=0.005) than the abnormal group. However, after treatment, the difference in the ratio of forward motile sperm between the two groups was no longer significant (*P*=0.112).

**Table 3:** Subgroup analysis stratified by fructose level, mean ± SD (median).

Parameters	<0.87 g/L (n=39)	≥0.87 g/L (n=184)	P
Basic information			
Age, year	34.03 ± 4.76 (33.00)	33.28 ± 4.48 (33.00)	0.514
Seminal plasma biochemical markers			
Fructose, g/L	0.54 ± 0.24 (0.58)	2.32 ± 0.92 (2.17)	<0.001
Zinc in seminal plasma, mmol/L	3.15 ± 0.89 (3.10)	2.31 ± 0.94 (2.18)	<0.001
Citric acid, umol/per time	102.22 ± 56.53 (97.68)	104.13 ± 58.58 (91.59)	0.947
Neutral glycosidase, mU/per time	63.55 ± 45.94 (56.70)	60.49 ± 40.58 (54.30)	0.733
Pretreatment semen parameters			
Abstinence, day	4.44 ± 1.37 (4.00)	4.06 ± 2.03 (4.00)	0.047
Ratio of forward motile sperm, %	19.37 ± 9.58 (17.20)	25.79 ± 12.85 (24.25)	0.005
Semen volume, ml	2.44 ± 1.09 (2.40)	3.51 ± 1.31 (3.40)	<0.001
Sperm concentration, million/ml	100.81 ± 115.71 (84.80)	50.84 ± 39.50 (40.75)	<0.001
Ratio of normal morphology, %	2.97 ± 2.08 (2.00)	2.58 ± 1.52 (2.00)	0.695
Total sperm count, million	196.75 ± 127.19 (193)	169.11 ± 130.97 (135.20)	0.072
Count of forward motile sperm, million	41.01 ± 38.68 (31.35)	42.37 ± 38.45 (32.02)	0.887
Posttreatment semen parameters			
Abstinence, day	4.21 ± 1.15 (4.00)	4.14 ± 1.29 (4.00)	0.639
Ratio of forward motile sperm, %	27.87 ± 13.20 (24.50)	31.06 ± 13.58 (30.40)	0.112
Semen volume, ml	2.54 ± 1.30 (2.20)	3.75 ± 1.34 (3.60)	<0.001
Sperm concentration, million/ml	121.37 ± 149.45 (94)	57.09 ± 46.50 (41.45)	<0.001
Ratio of normal morphology, %	3.67 ± 2.38 (3.00)	2.98 ± 1.64 (3.00)	0.117
Total sperm count, million	241.98 ± 214.34 (183.18)	199.75 ± 159.91 (155.61)	0.173
Count of forward motile sperm, million	65.75 ± 57.00 (51.24)	60.12 ± 53.16 (47.62)	0.564

The comparisons were using Student’s independent t test or Mann–Whitney U test (if normality assumptions were not met).

In the subgroup analyses stratified by zinc levels in seminal plasma, patients with abnormal zinc levels (<1.2mmol/L) exhibited significantly lower citric acid levels (*P*<0.05, **Table 4**).

**Table 4:** Subgroup analysis stratified by zinc level, mean ± SD (median).

Parameters	<1.2 mmol/L (n=22)	≥1.2 mmol/L (n=201)	P
Basic information			
Age, year	33.18 ± 4.19 (33.50)	33.43 ± 4.57 (33.00)	0.877
Seminal plasma biochemical markers			
Fructose, g/L	2.47 ± 1.44 (2.15)	1.96 ± 1.02 (2.00)	0.217
Zinc in seminal plasma, mmol/L	0.85 ± 0.27 (0.91)	2.63 ± 0.86 (2.56)	<0.001
Citric acid, umol/per time	71.44 ± 50.46 (61.56)	107.34 ± 57.90 (97.68)	0.001
Neutral glycosidase, mU/per time	73.51 ± 37.88 (70.02)	59.66 ± 41.71 (54.06)	0.066
Pretreatment semen parameters			
Abstinence, day	3.68 ± 1.43 (3.00)	4.17 ± 1.98 (4.00)	0.134
Ratio of forward motile sperm, %	29.14 ± 12.57 (27.15)	24.18 ± 12.50 (22.50)	0.062
Semen volume, ml	3.88 ± 1.48 (3.450)	3.26 ± 1.31 (3.20)	0.092
Sperm concentration, million/ml	56.23 ± 49.85 (39.60)	59.94 ± 64.12 (46.90)	0.650
Ratio of normal morphology, %	2.98 ± 1.78 (3.00)	2.62 ± 1.62 (2.00)	0.239
Total sperm count, million	177.20 ± 107.75 (160.13)	173.59 ± 132.93 (137.08)	0.507
Count of forward motile sperm, million	52.96 ± 41.48 (44.71)	40.94 ± 37.98 (29.54)	0.128
Posttreatment semen parameters			
Abstinence, day	4.09 ± 1.23 (4.00)	4.15 ± 1.27 (4.00)	0.834
Ratio of forward motile sperm, %	33.02 ± 11.28 (32.35)	30.22 ± 13.76 (29.00)	0.159
Semen volume, ml	3.70 ± 1.55 (3.20)	3.52 ± 1.39 (3.40)	0.843
Sperm concentration, million/ml	61.72 ± 55.47 (45.95)	69.05 ± 80.98 (49.70)	0.833
Ratio of normal morphology, %	2.98 ± 1.58 (3.00)	3.12 ± 1.83 (3.00)	0.898
Total sperm count, million	196.15 ± 137.73 (149.58)	208.34 ± 174.38 (162.84)	0.958
Count of forward motile sperm, million	62.97 ± 51.19 (45.38)	60.90 ± 54.15 (49.46)	0.707

The comparisons were using Student’s independent t test or Mann–Whitney U test (if normality assumptions were not met).

### Subgroup analyses stratified by seminal citric acid and neutral glycosidase levels

All variables were compared between the normal and abnormal groups for citric acid (**Table 5**) and neutral glycosidase (**Table 6**) levels in the seminal plasma. Patients with abnormal citric acid levels (<52 μmol/per time) had significantly lower levels of zinc and neutral glycosidase, reduced semen volume (pre and post), and a higher ratio of normal morphology (pre) (all *P*<0.05, **Table 5**). Additionally, the ratio of normal morphology was significantly higher in the abnormal citric acid group before treatment (*P*=0.012), but no significant difference was observed between the groups after treatment (*P*=0.478).

**Table 5:** Subgroup analysis stratified by citric acid level, mean ± SD (median).

Parameters	<52 umol/per time (n=40)	≥52 umol/per time (n=183)	P
Basic information			
Age, year	33.78 ± 4.41 (34.00)	33.33 ± 4.56 (33.00)	0.496
Seminal plasma biochemical markers			
Fructose, g/L	1.78 ± 0.91 (1.78)	2.06 ± 1.11 (2.04)	0.196
Zinc in seminal plasma, mmol/L	1.89 ± 0.95 (1.77)	2.58 ± 0.95 (2.56)	<0.001
Citric acid, umol/per time	35.72 ± 10.89 (35.37)	118.68 ± 53.40 (110.23)	<0.001
Neutral glycosidase, mU/per time	44.59 ± 36.19 (36.52)	64.62 ± 41.78 (57.12)	0.003
Pretreatment semen parameters			
Abstinence, day	3.93 ± 1.25 (4.00)	4.17 ± 2.05 (4.00)	0.679
Ratio of forward motile sperm, %	23.92 ± 11.92 (22.20)	24.83 ± 12.73 (23.80)	0.690
Semen volume, ml	2.54 ± 1.11 (2.35)	3.49 ± 1.33 (3.40)	<0.001
Sperm concentration, million/ml	85.67 ± 119.00 (51.05)	53.87 ± 39.87 (44.50)	0.318
Ratio of normal morphology, %	3.25 ± 1.78 (3.00)	2.52 ± 1.58 (2.00)	0.012
Total sperm count, million	162.64 ± 136.31 (134.07)	176.42 ± 129.39 (139.36)	0.384
Count of forward motile sperm, million	37.22 ± 36.72 (27.23)	43.20 ± 38.78 (32.16)	0.419
Posttreatment semen parameters			
Abstinence, day	4.05 ± 1.62 (4.00)	4.17 ± 1.18 (4.00)	0.357
Ratio of forward motile sperm, %	32.11 ± 16.08 (31.85)	30.15 ± 12.94 (29.20)	0.603
Semen volume, ml	2.84 ± 1.18 (2.65)	3.69 ± 1.41 (3.50)	<0.001
Sperm concentration, million/ml	99.08 ± 150.30 (50.80)	61.61 ± 49.74 (47.20)	0.406
Ratio of normal morphology, %	3.39 ± 2.13 (3.00)	3.04 ± 1.73 (3.00)	0.261
Total sperm count, million	199.33 ± 172.73 (136.60)	208.84 ± 170.91 (165.36)	0.478
Count of forward motile sperm, million	60.53 ± 54.95 (46.20)	61.23 ± 53.65 (49.51)	0.715

The comparisons were using Student’s independent t test or Mann–Whitney U test (if normality assumptions were not met).

**Table 6:** Subgroup analysis stratified by neutral glycosidase level, mean ± SD (median).

Parameters	<20 mU/per time (n=39)	≥20 mU/per time (n=184)	P
Basic information			
Age, year	33.44 ± 4.39 (33.00)	33.40 ± 4.57 (33.00)	0.938
Seminal plasma biochemical markers			
Fructose, g/L	1.94 ± 0.93 (2.00)	2.02 ± 1.11 (2.00)	0.953
Zinc in seminal plasma, mmol/L	2.68 ± 1.00 (2.65)	2.40 ± 0.97 (2.33)	0.093
Citric acid, umol/per time	71.63 ± 47.67 (55.23)	110.61 ± 57.94 (104.93)	<0.001
Neutral glycosidase, mU/per time	12.57 ± 4.76 (13.86)	71.30 ± 38.42 (63.32)	<0.001
Pretreatment semen parameters			
Abstinence, day	3.95 ± 1.23 (4.00)	4.16 ± 2.05 (4.00)	0.686
Ratio of forward motile sperm, %	25.31 ± 13.19 (22.50)	24.53 ± 12.46 (23.70)	0.803
Semen volume, ml	2.56 ± 1.14 (2.40)	3.48 ± 1.32 (3.40)	<0.001
Sperm concentration, million/ml	58.05 ± 114.24 (27.30)	59.90 ± 45.49 (48.30)	0.004
Ratio of normal morphology, %	2.63 ± 1.68 (2.50)	2.66 ± 1.63 (2.00)	0.887
Total sperm count, million	97.17 ± 82.23 (74.80)	190.22 ± 133.11 (154.23)	<0.001
Count of forward motile sperm, million	22.83 ± 20.63 (15.88)	46.22 ± 40.06 (34.44)	<0.001
Posttreatment semen parameters			
Abstinence, day	4.15 ± 1.53 (4.00)	4.15 ± 1.21 (4.00)	0.909
Ratio of forward motile sperm, %	30.42 ± 14.58 (29.30)	30.52 ± 13.35 (30.25)	0.786
Semen volume, ml	2.96 ± 1.33 (3.00)	3.66 ± 1.40 (3.50)	0.010
Sperm concentration, million/ml	74.18 ± 144.32 (37.90)	67.09 ± 56.51 (51.15)	0.192
Ratio of normal morphology, %	2.56 ± 1.14 (2.50)	3.22 ± 1.90 (3.00)	0.066
Total sperm count, million	135.18 ± 94.39 (113.19)	222.39 ± 179.55 (169.13)	0.003
Count of forward motile sperm, million	42.82 ± 36.86 (30.86)	64.98 ± 56.01 (51.00)	0.011

The comparisons were using Student’s independent t test or Mann–Whitney U test (if normality assumptions were not met).

As shown in **Table 6**, patients with abnormal neutral glycosidase levels (<20 mU/per time) had significantly lower citric acid levels, semen volume (pre and post), sperm concentration (pre), total sperm count (pre and post), and forward motile sperm count (pre and post) (all *P*<0.05). The sperm concentration was significantly lower in the abnormal neutral glycosidase group before treatment (*P*=0.004), but no significant difference was observed between the groups after treatment (*P*=0.192).

### Changing difference and changing ratio (%)

To mitigate the influence of baseline characteristics, the change in difference and change in ratio (%) of semen parameters were further analyzed. The change in difference was defined as the posttreatment results minus the pretreatment results, and the change in ratio (%) was defined as the ratio of the change in difference compared to the pretreatment results. As shown in **Table 7**, the changing of forward motile sperm ratio, sperm concentration, semen volume, ratio of normal morphology, total sperm count, and count of forward motile sperm all exhibited improvements after treatment in terms of changing differences (all *P*<0.01). Moreover, in the changing ratio (%), all nine parameters demonstrated positive improvements after treatment (all *P*<0.01).

**Table 7:** The changing difference and changing ratio (%) of semen parameters after treatment of all patients.

Parameters	Changing difference		Changing ratio (%)	
	Compared to 0	P	Compared to 0	P
Abstinence, day	0.02 ± 2.14	0.876	13.16 ± 52.93	<0.001
Ratio of forward motile sperm, %	5.83 ± 12.17	<0.001	46.53 ± 94.47	<0.001
Semen volume, ml	0.22 ± 0.97	<0.001	11.79 ± 37.02	<0.001
Sperm concentration, million/ml	8.75 ± 39.73	0.001	37.31 ± 105.53	<0.001
Ratio of normal morphology, %	0.45 ± 1.72	<0.001	42.99 ± 103.03	<0.001
Total sperm count, million	33.19 ± 139.67	<0.001	63.38 ± 177.58	<0.001
Count of forward motile sperm, million	18.98 ± 47.05	<0.001	130.14 ± 275.50	<0.001

The statistical test was one-sample t-test.

### Correlation coefficient and multiple linear regression

The correlations between changing difference or changing ratio (%) of semen parameters and the four seminal plasma biochemical markers were analyzed. As shown in **Table 8**, except for abstinence day, and ratio of normal morphology, all other semen parameters exhibited correlations with some of the seminal plasma biochemical markers. Notably, the ratio of forward motile sperm was negatively related to fructose (both changing difference and ratio, r=-0.165 and -0.144), and semen volume showed a negative correlation with neutral glycosidase (both changing difference and ratio, r=-0.158 and -0.178). In terms of changing ratio, it was found that semen volume, sperm concentration, total sperm count, and forward motile sperm count were all negatively related to citric acid and neutral glycosidase (r value range from -0.114 to -0.224). To further validate treatment improvement, a multiple linear regression analysis was conducted. After adjusting for the effects of fructose, zinc in seminal plasma, citric acid, neutral glycosidase levels, and patient age, only the ratio of forward motile sperm (adjusted changing difference=5.83, *P*=0.017) and ratio of normal morphology (adjusted changing difference=0.45, P=0.004) remained statistically significant. An interaction effect between the ratio of forward motile sperm and fructose level was also observed (*P*=0.006). In summary, based on the results of the changing ratio (%) and multiple linear regression, it appears that the improved ratio of forward motile sperm and ratio of normal morphology were influenced not only by seminal plasma biochemical markers or patient age.

**Table 8:** Correlation coefficient analysis between semen parameters’ posttreatment changing difference/ratio and seminal plasma biochemical markers.

Pearson’s r	Seminal plasma biochemical markers				
	Fructose	Zinc in seminal plasma	Citric acid	Neutral glycosidase	Age
Changing difference					
Abstinence, day	0.025	-0.069	-0.087	0.018	-0.095
Ratio of forward motile sperm, %	-0.165*	-0.026	-0.035	-0.023	-0.032
Semen volume, ml	0.030	-0.007	-0.024	-0.158*	0.108
Sperm concentration, million/ml	-0.112	0.072	-0.115	-0.066	0.061
Ratio of normal morphology, %	-0.109	0.018	0.029	0.097	-0.171*
Total sperm count, million	-0.037	-0.003	-0.070	-0.033	0.104
Count of forward motile sperm, million	-0.110	-0.030	-0.073	-0.037	0.082
Changing ratio (%)					
Abstinence, day	0.059	-0.073	-0.032	0.036	-0.100
Ratio of forward motile sperm, %	-0.144*	0.015	-0.035	-0.043	-0.006
Semen volume, ml	-0.009	0.007	-0.114	-0.178**	0.129
Sperm concentration, million/ml	0.054	-0.022	-0.151*	-0.180**	-0.014
Ratio of normal morphology, %	-0.007	0.020	0.014	0.047	-0.096
Total sperm count, million	0.057	-0.013	-0.178**	-0.224**	0.014
Count of forward motile sperm, million	-0.014	-0.006	-0.206**	-0.216**	0.066

The association between variables was analyzed using Pearson’s correlation coefficient.

*P<0.05; **P<0.01.

## Discussion

Free carnitine is predominantly present in epididymal tissue and semen (21). The epididymis serves as a site for sperm maturation and storage, where levels of free carnitine directly impact the processes of sperm maturation and metabolism, ultimately affecting sperm motility and fertilization ability. In this study, following the 3-month L-carnitine treatment, all semen parameters exhibited significant improvement in both difference and ratio (all *P*<0.01). These findings indicate that L-carnitine has a notable therapeutic effect on the overall enhancement of semen quality. This effect may be attributed to the fact that, as an effective antioxidant, L-carnitine can prevent the generation of reactive oxygen species (ROS) and promote their elimination, thereby safeguarding sperm from oxidative damage (22). In addition, L-carnitine functions as a mitochondrial protective agent by regulating the acetyl-CoA/CoA ratio and preventing ROS-mediated cell apoptosis, among other functions (23). However, optimal sperm function and fertilization require moderate levels of oxidative stress, but excessive oxidative stress can result in sperm damage and infertility (24). Moreover, patients with idiopathic oligoasthenoteratospermia may exhibit varying degrees of oxidative stress damage (25). While antioxidant drugs may be necessary for treatment, variations in drug absorption and utilization exist among patients, and the appropriate levels of oxidative stress for different epididymal environments still need investigation. Therefore, the presence of oxidative stress damage alone cannot be considered an indication for L-carnitine treatment.

In this study, the duration of abstinence was positively correlated with the level of seminal citric acid (r = 0.125*, *P* < 0.05), a marker indicative of the secretory function of the prostate gland (26). Therefore, a prolonged period of abstinence led to an elevated accumulation of citric acid in the prostate gland. The proportion of forward motile sperm exhibited a positive correlation with seminal fructose (r=0.275, *P*<0.01), a sugar secreted by the seminal vesicles known to provide the necessary energy for sperm motility (27). In contrast, the proportion of forward motile sperm exhibited a negative correlation with seminal zinc (r=-0.201, *P*<0.01), Zinc, primarily secreted by the prostate gland, has been associated with various critical processes that contribute to the acquisition of fertilization ability by spermatozoa, such as motility, capacitation, and acrosomal exocytosis (28). However, it should be noted that elevated levels of zinc in seminal plasma lead to an increase in intraspermatozoal zinc levels. Given the negative correlation between zinc and pH, an elevation in seminal plasma zinc may result in a reduction of intracellular pH. This pH decrease contributes to a decline in the respiration and motility of spermatozoa (29). Optimal concentrations of Zn2+ have been shown to enhance sperm motility, while elevated concentrations of zinc have been observed to hinder sperm motility (30).

Semen volume is associated with the secretory function of the accessory gonads, including the prostate gland and seminal vesicles (31). Seminal citric acid and seminal fructose serve as indicators of the secretory function of the prostate gland and seminal vesicles, respectively (26). Given that the secretions from these two glands constitute the primary proportion of semen volume, semen volume exhibited a positive correlation with seminal citric acid (r=0.543, *P*<0.01) and seminal fructose (r=0.392, *P*<0.01). Consistent with these findings, subgroup analyses stratified by seminal fructose showed that in both pretreatment and posttreatment, the normal fructose group exhibited a significantly higher semen volume than the abnormal fructose group. Conversely, an elevated semen secretion volume results in a reduction in sperm concentration, establishing a negative correlation between sperm concentration and seminal citric acid (r=-0.137, *P*<0.05)/seminal fructose (r=-0.344, *P*<0.01). Subgroup analyses stratified by seminal fructose also showed that in both pretreatment and posttreatment, the normal fructose group showed significantly lower sperm concentrations than the abnormal fructose group (both *P*<0.001). This observation can be attributed to the fact that when there is no significant difference in total sperm count between the two groups (pretreatment and posttreatment, both *P*>0.05), a high semen volume can lead to a decrease in sperm concentration.

Our findings demonstrated that sperm concentration was positively correlated with seminal zinc (r=0.152, *P*<0.01) emphasizing the crucial role of zinc in spermatogenesis. Zinc acts as a constituent of steroid receptors and metalloenzymes involved in DNA transcription (32). In this study, seminal neutral glycosidase was positively correlated with sperm concentration (r=0.155, *P*<0.01) and total sperm count (r=0.395, *P*<0.01). Seminal neutral glucosidase serves as a crucial marker of epididymal function (33). Given the pivotal role of the epididymis in sperm maturation and storage, the secretory function of the epididymis significantly influences both sperm concentration and quantity (34). In the subgroup analysis, the abnormal zinc group showed a significant decrease in citric acid concentration compared to the normal zinc group (*P*=0.001). This observation can be attributed to the roles of both zinc and citric acid in the secretion function of the prostate gland (35). Consequently, when the secretion function of the prostate gland diminishes, there is a certain degree of synchronicity between zinc and citric acid levels. Similarly, the subgroup analysis showed that in both pretreatment and posttreatment, the normal citrate acid group exhibited significantly higher semen volume than the citrate acid abnormal group (both *P*<0.001), as prostatic fluid constitutes the primary component of semen volume.

In this study, subgroup analyses revealed that some semen parameters exhibited significant differences between the two groups before treatment. Specifically, before treatment, the normal fructose group exhibited a significantly higher ratio of forward motile sperm than the abnormal fructose group (*P*=0.005). However, after three months of L-carnitine treatment, both groups showed improvement, and there was no significant difference in the ratio of forward motile sperm between the two groups. These findings suggest that L-carnitine may exert a more favorable therapeutic effect on the sperm vitality of the abnormal fructose group. Similarly, before treatment, the citric acid normal group displayed a lower proportion of normal sperm morphology than the citric acid abnormal group (*P*=0.012). However, after treatment, there was no significant difference between the two groups (*P*=0.261). These results imply that L-carnitine may have a more effective therapeutic effect on improving sperm morphology in the normal citric acid group. Moreover, before treatment, the abnormal neutral glycosidase group exhibited a significantly lower sperm concentration than the normal group (*P*=0.004). However, after treatment, there was no significant difference in sperm concentration between the two groups (*P*=0.192). These findings imply that L-carnitine may possess a stronger therapeutic potential for enhancing sperm concentration in the neutral glycosidase abnormal group. Furthermore, these findings also imply that these seminal plasma biochemical markers could potentially serve as biomarkers for determining indications for L-carnitine therapy in patients with idiopathic sperm abnormalities. Therefore, further investigations are needed to explore this potential.

In this study, the correlation analysis between the changing difference/ratio of semen parameters posttreatment and seminal plasma biochemical markers demonstrated a negative correlation between both the percentage increase and the rate of forward motility sperm with seminal fructose levels. Integrating this with the correlation analysis of seminal plasma biochemistry and pretreatment semen parameters, it is evident that seminal fructose plays a role in providing the energy needed for sperm activity in semen, as indicated by the positive correlation with the proportion of forward motile sperm. Nevertheless, there might be other related factors contributing to abnormal sperm motility in the normal fructose group, such as abnormal sperm tail shape and mitochondrial function. These factors could potentially influence the treatment efficiency of L-carnitine for these patients. Other studies have indicated no significant correlation between sperm vitality and seminal fructose concentration (19). However, further analysis found that the “Corrected Fructose (logarithm of sperm concentration multiplied by seminal fructose)” exhibits a significant positive correlation with sperm vitality (19).

The seminal neutral glucosidase levels exhibited negative correlations with the changing difference (r=-0.158, *P*<0.05) and changing ratio (r=-0.178, *P*<0.01) of semen volume, as well as the changing ratio of sperm concentration (r=-0.180, *P*<0.01), total sperm count (r=-0.224, *P*<0.01) and forward motility sperm count (r=-0.216, *P*<0.01). Seminal neutral glycosidase levels serve as indicators of the functional state of the epididymis, where sperm undergo further maturation, are stored, and aged sperm are absorbed. Abnormalities in the epididymis’s secretion function can lead to damage in the sperm storage environment, resulting in premature aging of the sperm. This, in turn, can impact both the concentration and quantity of sperm. The levels of L-carnitine in the epididymis directly influence the maturation and metabolic processes of sperm; therefore, L-carnitine supplementation proves particularly beneficial for patients with abnormal epididymal function.

In the subgroup analyses, neutral glycosidase levels were significantly higher in the normal citric acid group than in the abnormal citric acid group (*P*=0.003), while citric acid levels were significantly higher in the normal neutral glucosidase group than in the abnormal neutral glucosidase group (*P*<0.001). These results indicated that in patients with idiopathic oligoasthenoteratospermia, the secretion of neutral glycosidase and citric acid exhibits a certain degree of synchronization. In this study, the seminal citric acid levels were also negatively correlated with the changing ratio of sperm concentration (r=-0.151, *P*<0.05), total sperm count (r=-0.178, *P*<0.01), and forward motility sperm count (r=-0.206, P<0.01). This finding differs from previous research on the correlation between citric acid concentration and sperm parameters (26). Notably, our study found that the pretreatment and posttreatment sperm concentration, total sperm count, and forward motility sperm count were not significantly different between the normal citric acid group and the abnormal citric acid group. The underlying mechanism might be attributed to the synchronization in the secretion levels of neutral glycosidase and citric acid. Consequently, seminal citric acid levels may offer limited predictive value for assessing the therapeutic efficacy of L-carnitine.

The multivariate linear regression analysis, adjusted for the four seminal plasma biochemical markers, demonstrated that the changing ratio of forward motile sperm (adjusted changing difference=5.83, *P*=0.017) and normal morphology (adjusted changing difference=0.45, *P*=0.004) remained significant. These findings indicated the existence of potential additional predictors of L-carnitine treatment efficacy. Another plausible explanation is that L-carnitine might enhance sperm quality, specifically motility and morphology, through alternative mechanisms, such as protection against oxidative stress damage.

This study has certain limitations, including its single-center nature and a relatively small sample size. Another limitation is that the study did not consider other factors that may affect seminal plasma biochemical markers and semen parameters, such as age, body mass index, seminal ROS, seminal L-carnitine levels and lifestyle factors. In addition, additional subgroup analysis based on abstinence time could provide a more comprehensive and accurate assessment of the study results. Therefore, future studies should involve larger sample sizes and a more comprehensive range of variables and should be conducted in multiple centers to confirm the findings of this study. Furthermore, L-carnitine levels in seminal plasma were not assessed due to the lack of a universally recognized clinical testing assay kit.

## Conclusion

This study suggests that L-carnitine therapy effectively improved most semen parameters in patients with idiopathic sperm abnormalities. Seminal plasma biochemical markers, especially fructose, and neutral glycosidase, may be useful for determining indications for L-carnitine therapy in patients with idiopathic oligoasthenoteratospermia. Specifically, the L-carnitine treatment regimen may be more appropriate for asthenospermia patients with low seminal fructose levels, as well as low semen volume and oligospermia patients with low seminal neutral glycosidase levels.

## Data availability statement

The original contributions presented in the study are included in the article/supplementary material. Further inquiries can be directed to the corresponding author.

## Ethics statement

The studies involving humans were approved by The Guangdong Provincial Hospital of Chinese Medicine Research Ethics Committee (Approval No: ZE2023-157-01). The studies were conducted in accordance with the local legislation and institutional requirements. The requirement of patient informed consent was waived in view of the retrospective nature of the study.

## Author contributions

QY: Data curation, Formal analysis, Validation, Visualization, Writing – original draft. RH: Data curation, Methodology, Writing – review & editing. YN: Data curation, Resources, Writing – review & editing. MJ: Formal analysis, Software, Writing – review & editing. JL: Formal analysis, Writing – review & editing. ZC: Data curation, Writing – review & editing. DY: Conceptualization, Funding acquisition, Project administration, Supervision, Writing – review & editing.
